# Insights into the Intersection of Biocide Resistance, Efflux Pumps, and Sequence Types in Carbapenem-Resistant *Acinetobacter baumannii*: A Multicenter Study

**DOI:** 10.3390/pathogens12070899

**Published:** 2023-06-30

**Authors:** Zeeshan Taj, Muhammad Hidayat Rasool, Mohsin Khurshid, Bilal Aslam, Muhammad Usman Qamar

**Affiliations:** Institute of Microbiology, Government College University Faisalabad, Faisalabad 38000, Pakistan; drzeeshantaj@gcuf.edu.pk (Z.T.); drbilalaslam@gcuf.edu.pk (B.A.); musmanqamar@gcuf.edu.pk (M.U.Q.)

**Keywords:** antibiotic resistance, biocide resistance, efflux pumps, Pakistan, antibiotic resistance genes

## Abstract

*Acinetobacter baumannii*, a pathogenic bacterium acquired in hospitals, causes diverse infections in humans. Previous studies have reported resistance among *A. baumannii* strains, potentially selecting multi-drug-resistant variants. In Pakistan, research has primarily focused on carbapenem-resistant *A. baumannii* (CRAB) strains, overlooking the investigation of efflux pumps (EPs) and biocide resistance. This study aims to assess *A. baumannii* strains from five hospitals in Pakistan, focusing on antibiotic and biocide susceptibility, the impact of EP inhibitors on antimicrobial susceptibility, and the distribution of ARGs and STs. A total of 130 non-repeated *Acinetobacter baumannii* isolates were collected from five tertiary care hospitals in Pakistan and identified using API 20NE and multiplex PCR. Antimicrobial susceptibility testing utilized disc diffusion and broth microdilution assays, while biocide susceptibility was assessed with various agents. The impact of an efflux pump inhibitor (NMP) on antibiotic susceptibility was evaluated. PCR screening for ARGs and EPGs was followed by DNA sequencing validation. MLST was performed using the Pasteur scheme. Most isolates demonstrated resistance to tested antibiotics, with varying levels of susceptibility to biocides. All isolates exhibited the intrinsic class D β-lactamase *bla*_OXA-51_, while acquired *bla*_OXA-23_ was present in all CRAB isolates. Among EPs, *adeJ*, *abeD*, *amvA*, and *aceI* were prevalent in almost all isolates, with *adeB* found in 93% of isolates and *adeG*, *adeT1*, *adeT2*, and *qacEΔ1* displaying lower prevalence ranging from 65% to 79%. The most common STs were ST589 and ST2, accounting for 28.46% and 25.38% of isolates, respectively, followed by ST642 at 12.6%. These findings indicate that *A. baumannii* strains in Pakistan are resistant to antibiotics (excluding colistin and tigecycline) and may be developing biocide resistance, which could contribute to the selection and dissemination of multi-drug-resistant strains.

## 1. Introduction

*Acinetobacter baumannii* is a significant human pathogen causing diverse infections. The rise of antibiotic-resistant strains reduces treatment effectiveness and increases mortality rates. Comparative genomic studies have identified virulence factors associated with antibiotic resistance, host–pathogen interactions, immune evasion, and environmental persistence [1]. *A. baumannii* is a significant health threat. It can endure and inhabit different surroundings and exhibits resistance to numerous antimicrobial agents like antibiotics and biocides [2]. As a result, managing the spread and infection of *A. baumannii* in healthcare environments is a difficult task.

Biocides play a crucial role in limiting the spread of pathogenic microorganisms in hospitals, laboratories, industries, and households [3]. However, there is a growing concern about reduced susceptibility to biocides among bacterial species, including *A. baumannii*, in patients [4]. Research studies have consistently shown that *A. baumannii* isolates originating from hospitals exhibit lowered susceptibility to commonly used biocides such as hydrogen peroxide, formaldehyde, alcohols, chlorhexidine, iodophors, iodine, triclosan, and benzalkonium [5,6]. The extensive use of biocides raises concerns about a potential reduction in susceptibility and the emergence of multi-drug-resistant (MDR) pathogens [7]. Poor sanitation and exposure to sub-lethal concentrations of biocides may contribute to the persistence of *A. baumannii* in clinical settings [8,9]. Previous reports have also highlighted the cross-resistance between biocide exposure and antibiotic resistance in bacteria [10,11]. 

Efflux pumps (EPs) in bacteria are crucial mechanisms that contribute to reduced susceptibility to both antibiotics and biocides. Broad substrate EPs can remove numerous unrelated antibiotics and biocides from bacterial cells, which ultimately contributes to the multi-drug-resistant (MDR) strains [12]. *A. baumannii* possesses a considerable number of EPs with a wide substrate range that is associated with the inherent resistance to agents with different structures [13]. The EPs that are known to cause resistance not only to conventional antibiotics but also the biocides are of several types, like resistance nodulation cell division (RND) superfamily, which includes the *ade*ABC, *ade*FGH, *ade*IJK, and *abeD* pumps, the *amvA* pump of the major facilitator superfamily (MFS), multi-drug and toxic compound extrusion (MATE) family, i.e., *abeM*, the small drug resistance (SMR) family, i.e., *qacE* and *qacEΔ1*, and the proteobacterial antimicrobial compound efflux (PACE) family, i.e., *aceI*.

The previous studies conducted in Pakistan have primarily focused on the emergence and dissemination of CRAB strains within clinical settings, lacking concurrent investigation of resistance mechanisms in clinical *A. baumannii* isolates. Additionally, there is a dearth of information regarding the presence of EPs in *A. baumannii* strains and their resistance to biocides. Therefore, this study aims to comprehensively assess and compare the sequence types (STs) of *A. baumannii* strains, investigate multiple EPs, and explore biocide resistance. 

## 2. Materials and Methods

### 2.1. Bacterial Strains

From January 2020 to July 2021, a total of 130 non-repeated *Acinetobacter baumannii* isolates were collected from five different tertiary care hospitals (TCHs) in Pakistan, including three centers in Lahore (Punjab province), and one center each in Islamabad (Capital territory) and Peshawar (Khyber Pakhtunkhwa province). The most common sources were tracheal secretions (n = 30, 23.08%), followed by blood (n = 25, 19.23%) and sputum (n = 21, 16.15%). Retrospectively reviewing the clinical records of subjected patients, it was determined that all 130 non-repeated *A. baumannii* isolates were acquired from hospital-acquired infections (HAIs), as the isolates were obtained after 48 h of hospital stay. Firstly, the identification of the isolates was made using API 20NE (bioMérieux, France), and their identity was later confirmed through a multiplex PCR test targeting both the *recA* gene fragment and the intergenic spacer region specific to *A. baumannii*. The isolates were preserved in a brain–heart infusion (BHI) broth with glycerol at a temperature of −80 °C and were grown on BHI agar before testing.

### 2.2. Antimicrobial Susceptibility Testing

To study the antimicrobial susceptibility profile of the isolates, both disc diffusion assays (DDA) and broth microdilution (BMD) assays were performed for a range of antimicrobial agents, including amikacin, gentamicin, imipenem, tobramycin, piperacillin-tazobactam, ampicillin-sulbactam, ceftazidime, cefepime, imipenem, meropenem (DDA only), doxycycline, ciprofloxacin, and trimethoprim-sulfamethoxazole. For tigecycline and colistin, only BMD assays were conducted. The results of both DDA and BMD assays were interpreted based on the guidelines set forth by the Clinical and Laboratory Standards Institute (CLSI) and the criteria established by the US Food and Drug Administration (FDA) for determining susceptibility to tigecycline. In contrast, *Escherichia coli* (ATCC-35218 and ATCC-25922) and *Pseudomonas aeruginosa* (ATCC-27853) were used as quality control [14].

### 2.3. Susceptibility to Biocides

The biocidal agents, including benzalkonium bromide (BKB), a cationic quaternary ammonium compound, a biguanide, chlorhexidine (CHD), and triclosan, were chosen due to common usage in the global healthcare system. As per CLSI recommendations, stock solutions were prepared by dissolving 1000 μg/mL of biocides in sterile distilled water. Stock solutions were further diluted in Mueller–Hinton broth (MHB) (Oxoid, UK) to reach final well concentrations of 256–0.5 µg/mL after adding the inoculum (50 µL). The microdilution plates were incubated for 16–20 h at 37 °C and were visually read using an inverted reading mirror. To avoid discrepancy, an ELISA plate reader was used. The lowest concentration of the biocide, which efficiently inhibited the growth of the isolate, was considered as minimum inhibitory concentration (MIC).

### 2.4. Addition of Efflux Pump Inhibitor (EPI)

The method was used to assess the impact of an efflux pump inhibitor, i.e., 1-(1-Naphthylmethyl)-piperazine (NMP), on antibiotic susceptibility. We evaluated the susceptibility of bacteria to different antibiotics, including amikacin, gentamicin, cefepime, imipenem, doxycycline, and ciprofloxacin, using a method called BMD assays. These assays were conducted both in the presence and absence of NMP at a concentration of 100 μg/mL, following guidelines from the CLSI that have been previously described [15]. A 4-fold or greater reduction in the MIC values after the addition of NMP was considered significant. Different concentrations of antibiotics were added to MHB, and NMP was subsequently added to the corresponding plate. The MIC for each antibiotic was estimated in the absence and presence of NMP. 

### 2.5. Screening of ARGs and EPGs

To screen for genes that encode different types of ARGs, PCR was conducted using primers mentioned in Table S1. These include the extended-spectrum beta-lactamase genes (ESBLs) such as *bla*_TEM_, *bla*_SHV_, and *bla*_CTXM_ carbapenemases such as *bla*_OXA23_, *bla*_OXA24_, *bla*_OXA48_, *bla*_OXA51_, *bla*_OXA58_, *bla*_IMP_, *bla*_VIM_, *bla*_SIM_, *bla*_GIM_, *bla*_SPM_, *bla*_NDM_, and *bla*_KPC_, and for the presence of IS*Aba1* upstream of *bla*_OXA-23_ and *bla*_OXA-51_. PCR was performed to determine the distribution of various EPGs, including *adeB*, *adeG*, *adeJ*, *abeD*, *adeT1*, *adeT2*, *abeM*, *tetA*, *tetB*, *amvA*, *aceI*, *qacE,* and *qacEΔ1* in clinical isolates of *A. baumannii*, using the primers listed in Table S1. The reaction mixture, containing 50 µL, consisted of 25 µL of 2× PCR Master Mix (Thermo Fisher Scientific, Waltham, MA, USA), 1 µL of each primer (10 µM), and 1.5 µL of the DNA sample. The PCR was carried out using a T100 Thermal Cycler (Bio-Rad Laboratories, Hercules, CA, USA) under the following conditions: denaturation at 95 °C for 3 min, followed by 35 cycles of 95 °C for 30 s, 50 °C for 30 s, and 72 °C for 30 s, and a final extension at 72 °C for 12 min. Afterward, DNA sequencing was done to validate the PCR products [16].

### 2.6. Multilocus Sequence Typing (MLST)

The Pasteur MLST scheme was followed to perform MLST on all the 130 *A. baumannii* isolates by amplifying seven housekeeping genes using specific primers (Table S1) synthesized from Shanghai Jieli Biotechnology Co., Ltd. (Shanghai, China). Briefly, a 50 µL reaction mixture was used for PCR, including 25 µL of 2× PCR Master Mix (Thermo Fisher Scientific, Waltham, MA, USA), 1 µL of each primer (10 µM), and 1.5 µL of sample DNA. The PCR was conducted in a T100 Thermal Cycler (Bio-Rad Laboratories, Hercules, CA, USA), with the following conditions: denaturation at 95 °C for 3 min, followed by 35 cycles of 95 °C for 30 s, 50 °C for 30 s, and 72 °C for 30 s, and a final extension at 72 °C for 12 min. The amplicons were analyzed by agarose gel electrophoresis (AGE) with 1.2% agarose gel at 90 volts for 35–40 min in 1× TAE buffer. The 100 bp plus DNA Marker (Thermo Fisher Scientific, Waltham, MA, USA) was used to determine the size of the amplicons. The amplified products were sequenced by the Sanger sequencing method. The sequences were subjected to analysis by means of the database (PubMLST) available at https://pubmlst.org/bigsdb?db=pubmlst_abaumannii_seqdef, accessed on 2 March 2023.

## 3. Results

### 3.1. Clinical Characteristics of A. baumannii Isolates

A total of 130 *A. baumannii* isolates were obtained, primarily from tracheal secretions (30 patients, 23.08%), followed by blood (25 patients, 19.23%), sputum (21 patients, 16.15%), and other specimens, as shown in Table 1. Most patients were admitted to the ICU (43 patients, 33.08%), with others admitted to medicine (29 patients, 22.31%), surgery (20 patients, 15.38%), and other departments. The age of patients varied, with the majority falling between 21 and 60 years old (57 patients, 43.85%), followed by those aged between 61 and 70 (36 patients, 27.69%), and those aged ≤20 (19 patients, 14.62%). These patients were admitted to five different TCHs, with Hospital A (located in Lahore, Punjab) and Hospital D (located in Islamabad) having the most patients (36 patients, 27.69%), followed by Hospitals B (32 patients, 24.62%), E (14 patients, 10.77%), and C (12 patients, 9.23%), located in Lahore, Multan (Punjab), and Peshawar (Khyber Pakhtunkhwa), respectively.

### 3.2. Susceptibility to Antibiotics and Biocides

In total, 14 antibiotics and 3 biocides were tested (Table 2) on all the isolates. Unfortunately, most of the isolates were found resistant to studied antibiotics, including β-lactams alone and in combination with β-lactamase inhibitors (such as ceftazidime, cefepime, imipenem, meropenem, ampicillin-sulbactam, and piperacillin-tazobactam) at a rate of 89.23%, as well as aminoglycosides (amikacin, gentamicin, and tobramycin) at rates ranging from 80.77% to 89.23%, tetracycline (doxycycline) at 83.08%, fluoroquinolones (ciprofloxacin) at 92.31%, and trimethoprim-sulfamethoxazole at 79.23%.

The isolates also showed varying levels of susceptibility to the three biocides tested. For triclosan, the estimated MICs range was from 2 to 64 μg/mL, with MIC50 and MIC90 values of 32 μg/mL and 64 μg/mL, respectively. CHD had a MIC range from 4 to 128 μg/mL, with MIC50 and MIC90 values of 64 μg/mL and 128 μg/mL, respectively, while BKB had a MIC range from 4 to 64 μg/mL, with MIC50 and MIC90 values of 16 μg/mL and 32 μg/mL, respectively. As clinical interpretative criteria are not available, it is difficult to categorize the isolates as susceptible or resistant to the tested biocides. However, the MIC distribution to biocides permitted the estimation of the susceptibility patterns for the isolates.

### 3.3. Effect of EPI on Antimicrobial Susceptibility

The study results indicated that the efflux pump inhibitor used in the experiment had a significant impact on the MIC of ciprofloxacin and doxycycline, while the changes in the MIC distribution of cefepime and imipenem were relatively minor with only a few isolates with four-fold reduction, and no significant (four-fold) changes were observed for amikacin and gentamicin. Out of the 130 clinical isolates of *A. baumannii* tested, 88 isolates showed a four-fold reduction in the MIC of doxycycline when the EPI was present, and 7 isolates showed an even greater (≥eight-fold) reduction. Similarly, for ciprofloxacin, the addition of the EPI resulted in a four-fold reduction in the MIC of 79 isolates, and 15 isolates showed an even greater (≥eight-fold) reduction, as presented in Table 3.

### 3.4. Distribution of ARGs

Overall, in screening, none of the (130 *A. baumannii)* isolates were found to have metallo-β-lactamase, but all were positive for the intrinsic class D β-lactamase, specifically *bla*_OXA-51_. Whereas, from acquired class D β-lactamases, only *bla*_OXA-23_ was detected in most of the carbapenem-resistant isolates (116/130, or 89.23%). In the 116 isolates with carbapenem resistance and *bla*_OXA-23_, the presence of IS*Aba1* was also observed upstream of the *bla*_OXA-23_ genes. 

Isolates were subjected to PCR-based screening to determine the presence of genes encoding aminoglycoside-modifying enzymes (AMEs) and 16SrRNA methylase. The *armA* was the sole 16s methylases gene that was found in 70 of the strains (53.85%), while other 16S methylase genes were not found in any *A. baumannii* isolates. Of the AME genes investigated, *aphA6*, *aadB*, *aacC1*, and *aphA1* were positive in 76.15% (n = 99), 66.15% (n = 86), 25.38% (n = 33), and 10% (n = 13) of the isolates, respectively. The *aadA1* gene was not detected in any of the isolates. Of the total isolates, 20 (15.38%) were found to possess the *sul1* gene, while the *sul2* gene was detected in 88 (67.69%) isolates.

### 3.5. Distribution of EPGs and BRGs

Overall, a total of 13 EPGs and BRGs were detected that encode efflux pumps belonging to different families. A significant proportion of these genes were present in most of the isolates. The genes *adeJ*, *abeD*, *amvA*, and *aceI* were present in 95–100% of *A. baumannii* isolates. The gene *adeB* was detected in 121 (93.08%) isolates. However, the prevalence of *adeG*, *adeT1*, *adeT2*, and *qacEΔ1* was lower, ranging from 65.38% to 79.23% (Table 4).

### 3.6. Multilocus Sequence Typing

A total of 156 isolates collected from five tertiary-care hospitals underwent MLST analysis, identifying 11 sequence types (STs). The two most common STs among the isolates were ST589 and ST2, accounting for 37 (28.46%) and 33 (25.38%) isolates, respectively. ST642 was the third most common type, found in 19 (12.6%) isolates. All isolates belonging to the three most common STs, as well as 8 (6.15%) and 6 (4.62%) isolates of ST1241 and ST600, respectively, and 5 (3.85%) isolates of both ST103 and ST889 and 3 (2.31%) isolates of ST615 were found to be resistant to carbapenems. On the other hand, 9 isolates of ST338, 4 isolates of ST708, and one isolate of ST597 were found to be susceptible to carbapenems.

### 3.7. Sequence Types-Specific Analysis of ARGs and EPGs 

The analysis of the distribution of various acquired resistance mechanisms has shown that different antimicrobial resistance determinants, particularly carbapenemases (*bla*_OXA-23_), 16S methylases (*armA*), and the *tetB* efflux gene, exhibit sequence type-specific distribution. However, aminoglycoside modifying enzymes (AMEs) were distributed randomly among the clones, particularly for the dominant sequence types (STs) ST589 and ST2. This study demonstrates that all isolates belonging to ST589 (corresponding to CC1) have *bla*_OXA23_ with an upstream IS*Aba1*, *armA* (16S methylase), and the *tetB* gene. Additionally, the AMEs *aphA6* and *aadB* were found in 25 and 23 isolates, respectively. A comprehensive overview of the distribution of acquired antimicrobial resistance genotypes and MIC ranges across different STs in *A. baumannii* isolates is provided in Table 5. 

## 4. Discussion

In clinical settings, *A. baumannii* is recognized as one of the significant pathogens due to its ability to persist in healthcare environments for extended periods [1]. Triclosan, a key compound in cleaning products such as hand sanitizers, toothpaste, and soaps, is also used for disinfecting medical tools [17]. Benzalkonium bromide is a cationic quaternary ammonium surfactant with antimicrobial properties commonly employed as a disinfectant in clinical settings. It functions by disrupting the lipid membrane bilayer of bacteria, causing leakage of cellular components, and exhibiting antibacterial activity [18]. Chlorhexidine, a biguanide antimicrobial, possesses broad-spectrum activity. It is frequently used as a topical antiseptic and for treating inflammatory dental conditions caused by microorganisms. Moreover, it is one of the most used antiseptic agents for skin and mucous membrane disinfection today [19]. Exposure to environmentally relevant levels of biocides may lead to the development of resistance in *A. baumannii* strains in the environment, which could contribute to the nosocomial dissemination of *A. baumannii*. Currently, there is a lack of global studies examining BRGs and susceptibility profiles in MDR *A. baumannii* strains [20]. This knowledge gap is particularly evident in Pakistan, where no such reports exist. Hence, the objective of this study was to assess the prevalence of BRGs in *A. baumannii*, establish a correlation with antimicrobial resistance phenotypes, and assess the presence of ARGs.

Earlier studies conducted in Pakistan have established a significant resistance rate (≥90%) of *A. baumannii* to cephalosporins and carbapenems. However, the resistance to carbapenems showed variation across different regions, with overall low MICs against carbapenems [21,22]. This disparity in resistance patterns could be attributed to variations in antimicrobial usage in different healthcare settings and the colonization of specific bacterial strains carrying class D carbapenemases, which exhibit lower hydrolytic efficacy for carbapenems compared to metallo-beta-lactamases (MBLs) [23]. In the present study, *bla*_OXA-23_ was detected in nearly all CRAB isolates. Previous research has indicated that the dissemination of *bla*_OXA-23_ is facilitated by mobile genetic elements (MGEs) such as plasmids, IS elements, and transposons. The current findings align with previous studies, revealing the presence of IS*Aba1* upstream of the *bla*_OXA-23_-like gene in all isolates. This genetic element is responsible for conferring carbapenem resistance, which is consistent with past research. Moreover, it has been observed that the expression of *bla*_OXA-23_-like genes in the presence of IS*Aba1* can impact the MICs of carbapenems for *A. baumannii* isolates [24,25]. 

Numerous studies have investigated antibiotic resistance in *A. baumannii*, and in recent years, the emergence of biocide-resistant strains has posed a significant clinical challenge. The prevalence of antibiotics and biocide-resistant *A. baumannii* in clinical settings has been extensively documented worldwide [5,26]. *A. baumannii* exhibits a higher rate of biofilm formation compared to other species, which contributes to resistance phenotypes, the development of the resistome, and the dissemination of resistance genes within biofilms through conjugation or transformation [27]. Although no statistically significant difference was observed, a study reported a numerical variation in biofilm-forming capacity between multi-drug-resistant (MDR) and non-MDR isolates. Additionally, there was a trend of meropenem-resistant isolates being the most proficient biofilm producers [28]. However, the relationship between antibiotic resistance and reduced biocide susceptibility remains unclear. This may be attributed to the use of clinical isolates from distinct genotypes and diverse healthcare facilities. Moreover, the studied antibiotics and biocides have different modes of action and target sites such as cell walls, proteins, nucleic acids, fatty acids, and membranes. The presence of resistance determinants encoding β-lactamases, AMEs, and broad-spectrum efflux pumps has been revealed in studies, indicating that these determinants confer resistance to both antibiotics and biocides in *A. baumannii* strains [4,29].

Multi-drug efflux pumps are frequently observed as a resistance mechanism to structurally analogous antibiotics, and they are significantly present in *A. baumannii* [30]. The biocide resistance mechanisms related to the outer membrane barrier that was previously proposed could also contribute to biocide resistance. Generally, Gram-negative pathogens show less susceptibility to biocides as compared to Gram-positive bacteria [30]. Therefore, the distribution and role of 13 drug efflux pumps of several families were assessed in this study. Overall, a significant prevalence of efflux pump genes was estimated in the tested isolates. The results indicated that *adeJ*, *abeD*, *amvA*, and *aceI* genes were detected in approximately all the isolates (95–100%). The *adeB* gene was found in 121 (93.08%) isolates. However, the prevalence of other genes, such as *adeG*, *adeT1*, *adeT2*, and *qacEΔ1*, was relatively lower, ranging from 65.38% to 79.23%. The *qacE* and *qacEΔ1* genes are frequently present in MGEs. However, they can be integrated into the chromosome depending on the evolution of specific resistance mechanisms [31]. In current findings, the MIC_90_ of CHD to *A. baumannii* was 128 µg/mL, which is higher than a large-scale study that reported the MIC_90_ to chlorhexidine gluconate as 25 µg/mL [32]. In another study, the MIC90 of chlorhexidine gluconate for *A. baumannii* isolates belonging to international clone (IC)-II was 100 µg/mL, and for non-IC-II isolates was <100 µg/mL [33]. In our study, the MIC_90_ value for triclosan was 64 μg/mL. A study from China reported that 2.7% of *A. baumannii* isolates had MIC ≥ 1 μg/mL [34]. A few studies have indicated that the efflux pumps *ade*ABC in *A. baumannii* transport the benzalkonium bromide and chlorhexidine. These results were also supported by a more recent investigation using *A. baumannii* ATCC 19606 and efflux pump mutants [9,35].

Various research studies conducted globally have shown that MLST clonal complexes CC2 (Pasteur scheme)/CC92 (Oxford scheme) are frequently involved in outbreaks. However, the situation in Pakistan is slightly different. Recent studies conducted in Pakistan, including this one, have reported the frequency of ST589 corresponding to CC1 (Pasteur scheme), with ST2 corresponding to CC also found in comparable numbers. Both ST589 and ST2 were prevalent in all hospitals, and they can be considered the most important CRAB clones, as per this and previous studies [22]. It is a well-established fact that isolates belonging to CC2 are commonly found worldwide because of their ability to selectively acquire resistance determinants and thrive in hospital environments, which gives them an advantage in being selected under antibiotic pressure [36]. 

All isolates belonging to the dominant STs (ST889, ST2, ST642, ST600) were found to have the *bla*_OXA-23_ gene. The results suggested a connection between the *bla*_OXA-23_ and the genetic background of CC1 and CC2, which gives the impression that it augments the carbapenem-resistant in *A. baumannii* strains. Studies have indicated that the distribution of the *bla*_OXA-23_ gene is linked to certain clones of *A. baumannii* worldwide, predominantly the international CC92. The CC92 of *A. baumannii* is regarded as the most extensive and widely distributed CC in the MLST database. Moreover, the *bla*_OXA-23_-harboring CRAB (CC92) has been well-reported from various parts of the globe, including the USA and China [24,37,38,39,40,41]. This suggests that this clonal complex has a wide geographic distribution and can spread to different regions. Moreover, *A. baumannii* CC2 carrying IS*Aba1* upstream to oxacillinases has been reported from Italy, Japan, Italy, and USA [37,38,41].

The study of the spread of different acquired mechanisms for resistance has revealed that certain genes, such as oxacillinases (*bla*OXA23), 16S methylases (*armA*), and the *tetB* efflux gene have ST-specific distributions. Molecular epidemiological studies have shown that CRAB outbreaks and spread are often caused by clonal dissemination. Certain strains, known as epidemic clones, can spread widely within and across countries, suggesting they possess traits that allow them to thrive in hospital environments and cause HAIs [39,42]. However, many studies lack an examination of the genetic differences between carbapenem-susceptible *A. baumannii* (CSAB) and CRAB. In our current study, we found that CSAB sequence types were infrequent, and most CRAB strains belonged to ST589, ST2, ST642, ST1241, ST600, ST103, and ST889, all of which harbored *bla*_OXA23_. In contrast, the distribution of AMEs does not appear to be linked with specific sequence types. This suggests that the acquisition and spread of AME genes is a relatively random process, driven by factors such as horizontal gene transfer and selective pressure rather than by specific genetic factors within bacterial populations. The studies have suggested that the transfer of genetic material between bacterial species contributes to the widespread dissemination of AMEs among clinical isolates of *A. baumannii,* which has become a serious health concern in recent years. These AMEs work by modifying aminoglycosides through phosphorylation, acetylation, or adenylation, thereby rendering the antibiotics ineffective [43]. The genes that encode these modifying enzymes possess the ability to be disseminated both within and between bacterial species as they are frequently located on MGEs. The mobility of these genetic elements allows the genes to spread rapidly and contribute to the development of antibiotic resistance. It is important to note that the spread of antibiotic resistance genes is a significant public health concern, as it limits the efficacy of antibiotics and poses a severe threat to the successful management of resistant bacterial pathogens and associated infections [44,45].

## 5. Conclusions

The present study investigated the molecular epidemiology of *A. baumannii* in Pakistan, revealing a sequence type-specific distribution of biocide resistance genes (BRGs), antibiotic resistance genes (ARGs), and resistance phenotypes. Among the *A. baumannii* isolates, the genes *adeJ*, *abeD*, *amvA*, and *aceI* were consistently present in 95–100%, while *adeB* was detected in 93.08% (121 isolates). The prevalence of *adeG*, *adeT1*, *adeT2*, and *qacEΔ1* varied from 65.38% to 79.23%. High resistance rates against cephalosporins and carbapenems were observed, predominantly associated with the presence of the *bla*_OXA-23_ gene in nearly all carbapenem-resistant *A. baumannii* (CRAB) isolates, which were genetically linked to clonal complexes CC1 and CC2. Notably, ST589 and ST2 emerged as major CRAB clones in Pakistan, deviating from the commonly reported CC2 clonal complex worldwide. These findings suggest the global dissemination of the *bla*_OXA-23_ gene through specific *A. baumannii* clones, particularly the internationally prevalent CC92, which highlights its potential for regional spread. Further investigations are warranted to explore the distribution of BRGs and susceptibility patterns among multidrug-resistant *A. baumannii* strains in different geographical regions.

## Figures and Tables

**Table 1:** Clinical characteristics of patients.

Gender	Number (Percentage)	Specimen Source	Number (Percentage)	Department	Number (Percentage)	Age	Number (Percentage)	Hospital	Number (Percentage)
Male	85 (65.38%)	Tracheal secretion	30 (23.08%)	Cardiology	1 (0.77%)	≤20	19 (14.62%)	A	36 (27.69%)
Female	45 (34.62%)	Blood	25 (19.23%)	ICU	43 (33.08%)	21–60	57 (43.85%)	B	32 (24.62%)
		Sputum	21 (16.15%)	Medicine	29 (22.31%)	61–70	36 (27.69%)	C	12 (9.23%)
		Urine	12 (9.23%)	Oncology	21 (16.15%)	>70	18 (13.85%)	D	36 (27.69%)
		Pus	15 (11.54%)	Pediatrics	8 (6.15%)			E	14 (10.77%)
		Wound swab	8 (6.15%)	Surgery	20 (15.38%)				
		Bronchial washings	6 (4.62%)	Urology	8 (6.15%)				
		CSF	4 (3.08%)						
		Endotracheal tube	4 (3.08%)						
		Catheter tip	4 (3.08%)						
		Fluid	1 (0.77%)						

**Table 2:** Susceptibility of *A. baumannii* isolates to antimicrobial agents and biocides.

Antimicrobial Agents	MIC (µg/mL)				
	Breakpoints	MIC_50_	MIC_90_	Susceptible (%)	Resistant (%)
Amikacin	≥64	512	1024	14 (10.77)	116 (89.23)
Gentamicin	≥16	256	512	17 (13.08)	113 (86.92)
Tobramycin	≥16	256	512	25 (19.23)	105 (80.77)
Piperacillin-Tazobactam	≥128/4	256	512	14 (10.77)	116 (89.23)
Ampicillin-Sulbactam	≥32/16	512	512	14 (10.77)	116 (89.23)
Ceftazidime	≥32	256	512	14 (10.77)	116 (89.23)
Cefepime	≥32	128	256	14 (10.77)	116 (89.23)
Imipenem	≥8	16	32	14 (10.77)	116 (89.23)
Meropenem	NT	-	-	14 (10.77)	116 (89.23)
Doxycycline	≥16	64	128	22 (16.92)	108 (83.08)
Tigecycline	≥8	1	1	130 (100)	0 (0)
Colistin	≥4	0.5	1	130 (100)	0 (0)
Ciprofloxacin	≥4	64	128	10 (7.69)	120 (92.31)
Trimethoprim-Sulfamethoxazole	≥4/76	32/608	64/1216	27 (20.77)	103 (79.23)
Triclosan	NA	32	64	-	-
Chlorhexidine Diacetate	NA	64	128	-	-
Benzalkonium Bromide	NA	16	32	-	-

**Table 3:** MIC values of antimicrobial agents against *A. baumannii* isolates with and without NMP addition.

Antimicrobial Agents		MIC (μg/mL)													Fold-Change in MIC (Number of Isolates)				
		0.125	0.25	0.5	1	2	4	8	16	32	64	128	256	≥512	2-Fold ↑	No Change	2-Fold ↓	4-Fold ↓	≥8-Fold ↓
Amikacin	Alone	-	-	-	-	1	10	3	-	-	4	25	16	71	12	68	50	-	-
	With NMP	-	-	-	-	5	8	1	-	-	11	28	18	59					
Gentamicin	Alone	-	-	-	9	8	-	-	2	14	7	17	29	44	14	58	57	1	-
	With NMP	-	-	4	12	1	-	-	14	3	15	11	35	35					
Cefepime	Alone	-	-	-	-	4	5	5	-	-	27	55	34	-	9	36	73	9	3
	With NMP	-	-	-	3	7	4	-	-	19	43	43	11	-					
Imipenem	Alone	-	-	6	8	-	-	10	56	50	-	-	-	-	9	22	96	3	-
	With NMP	-	4	8	2	-	-	55	49	12	-	-	-	-					
Doxycycline	Alone	-	-	4	15	3	-	-	1	21	53	33	-	-	-	-	35	88	7
	With NMP	3	5	12	2	-	4	13	47	44	-	-	-	-					
Ciprofloxacin	Alone	-	1	7	2	-	-	-	12	25	53	30	-	-	-	-	36	79	15
	With NMP	2	8	-	-	2	7	27	42	42	-	-	-	-					

**Table 4:** The distribution of EPGs and BRGs, as well as the MIC values of biocides across different sequence types of *A. baumannii*.

Sequence Types	Isolates n (%)	Gene Distribution															
		*adeB*	*adeG*	*adeJ*	*abeD*	*adeT1*	*adeT2*	*abeM*	*tetA*	*tetB*	*amvA*	*qacE*	*qacEΔ1*	*aceI*	TRI	CHD	BB
ST589	37 (28.46)	+	+	+	+	+	+	+	−	+	+	+	+	+	16–64	16–128	16–32
ST2	33 (25.38)	+	+	+	+	+	+	+	−/+	+	+	+	+	+	16–64	32–64	16–32
ST642	19 (14.62)	+	+	+	+	+	+	+	−	+	+	+	−	+	16–32	32–64	8–64
ST338	9 (6.92)	−	−	+	+	−	−	−	−	−	+	+	+	+	2–4	4–16	4–8
ST1241	8 (6.15)	+	+	+	+	−	−	+	−	+	+	−	−	+	16–64	16–32	8–16
ST600	6 (4.62)	+	+	+	+	−	+	+	−	+	+	+	+	+	16–32	32–64	16–32
ST103	5 (3.85)	+	−	+	+	−	−	+	−	+	+	−	−	+	16–32	16–32	8–16
ST889	5 (3.85)	+	−	+	+	+	−	+	−	−	+	−	−	+	4–8	8–16	4
ST708	4 (3.08)	+	−	+	+	−	−	−	−	−	−	−	−	+	4–8	16–64	8–16
ST615	3 (2.31)	+	−	+	+	+	+	+	−	−	+	−	−	+	8–16	32–64	8–16
ST597	1 (0.77)	+	−	+	−	−	−	−	−	−	−	−	−	−	4	8	4

**Table 5:** Sequence type-wise distribution of MIC and acquired antimicrobial-resistant genotypes in *A. baumannii* isolates.

Sequence Types	Total Number of Isolates	MIC (µg/mL) Range Except Those Specified											Acquired Antimicrobial-Resistant Genotypes (Number of Isolates)
		SAM	TZP	CAZ	FEP	IPM	AK	CN	TOB	DO	SXT	CIP	
ST589	37	≥512	256- ≥ 512	256- ≥ 512	128–256	16–32	≥512	256- ≥ 512	256- ≥ 512	32–64	32–64	64–128	*bla*_OXA-23_ (37), IS-*bla*_OXA-23_ (37), *armA* (37), *aphA6* (25), *aadB* (23), *tetB* (37), *sul2* (37)
ST2	33	256- ≥ 512	128–256	128–256	64–256	8–32	≥512	≥512	256- ≥ 512	64–128	16–64	32–128	*bla*_OXA-23_ (33), IS-*bla*_OXA-23_ (33), *armA* (33), *aphA1* (5), *aphA6* (28), *aacC1* (17), *aadB* (28), *tetA* (17), *tetB* (33), *sul1* (12), *sul2* (26)
ST642	19	256- ≥ 512	128–256	128–256	64–128	16–32	128–256	32–128	16–64	64–128	16–32	64–128	*bla*_OXA-23_ (19), IS-*bla*_OXA-23_ (19), *aphA6* (19), *aacC1* (11), *aadB* (19), *tetB* (19), *sul2* (19)
ST338	9	4–8	2–4	4–8	4–8	0.5–1	2–8	1–2	1–2	1–2	0.5–2	0.25–1	-
ST1241	8	256- ≥ 512	128–256	128–256	128–256	16–32	64–128	16–32	1–4	16–64	0.5–1	16–64	*bla*_OXA-23_ (8), IS-*bla*_OXA-23_ (8), *aphA1* (8), *aphA6* (8), *tetB* (8)
ST600	6	256- ≥ 512	128	128–256	128–256	16–32	256- ≥ 512	64–256	32–128	32–64	32–64	32–64	*bla*_OXA-23_ (6), IS-*bla*_OXA-23_ (6), *aphA6* (6), *aadB* (6), *tetB* (6), *sul2* (6)
ST103	5	256- ≥ 512	128	64–128	64–128	16–32	128–256	64–128	32–64	32–64	4–16	16–32	*bla*_OXA-23_ (5), IS-*bla*_OXA-23_ (5), *aphA6* (5), *aadB* (5), *tetB* (5), *sul1* (5)
ST889	5	128–256	128	64–128	64–128	16–32	64–256	128–256	32–128	0.5–1	0.5–1	16–32	*bla*_OXA-23_ (5), IS-*bla*_OXA-23_ (5), *aphA6* (5), *aacC1* (5), *aadB* (5)
ST708	4	4–8	2–4	2–8	2–4	0.5–1	4–8	2	2	0.5–1	0.5–1	16–32	-
ST615	3	64–128	128	64–128	64	8–16	64–128	1–2	1–2	1–2	4–8	16–32	*bla*_OXA-23_ (3), IS-*bla*_OXA-23_ (3), *aphA6* (3), *sul1* (3)
ST597	1	8	4	4	2	1	4	1	1	1	1	0.5	-

## Data Availability

The data presented in this study are available in this article or Supplementary Table S1.

## Supplementary Materials

The following supporting information can be downloaded at: https://www.mdpi.com/article/10.3390/pathogens12070899/s1, Table S1: Primers used in this study.
