# Supplementary material for: Insights into the Intersection of Biocide Resistance, Efflux Pumps, and Sequence Types in Carbapenem-Resistant Acinetobacter baumannii: A Multicenter Study
Source: Pathogens. 2023 Jun 30;12(7):899. doi: 10.3390/pathogens12070899 (PMC10383717; doi:10.3390/pathogens12070899)
Supplement: Supplementary file 1 [file pathogens-12-00899-s001.zip › pathogens-2440087-supplementary.pdf]

Table S1: Primers used in this study

Genes	Primers	Sequence (5′ to 3′)	Aimed Product (bp)	Annealing Temperature
recA	*rec*A-F	CCTGAATCTTCTGGTAAAAC	425	55
	*rec*A-R	GTTTCTGGGCTGCCAAACATTAC		
ITS region	ITS-1	CATTATCACGGTAATTAGTG	208	55
	ITS-2	AGAGCACTGTGCACTTAAG		
bla_{TEM}	TEM-F	TCAACATTTCCGTGTCG	860	56
	TEM-R	CTGACAGTTACCAATGCTTA		
bla_{SHV}	SHV-F	ATGCGTTATATTCGCCTGTG	896	56
	SHV-R	AGATAAATCACCACAATGCGC		
bla_{CTXM}	CTXMU-F	ATGTGCAGYACCAGTAARGT	593	52
	CTXMU-R	TGGGTRAARTARGTSACCAGA		
bla_{OXA-23}	23-F	ATGAATAAATATTTTACTTGCTATGTG	822	55
	23-R	TTAAATAATATTCAGCTGTTTTAATGA		
bla_{OXA-24}	24-F	GGTTAGTTGGCCCCCTTAAA	246	52
	24-R	AGTTGAGCGAAAAGGGGATT		
bla_{OXA-51}	51-F	ATGAACATTAAAGCACTCTTACTT	825	55
	51-R	CTATAAAATACCTAATTGTTCTAA		
bla_{OXA-58}	58-F	AAGTATTGGGGCTTGTGCTG	599	52
	48-R	CCCCTCTGCGCTCTACATAC		
bla_{OXA-48}	OXA-48-F	TTGGTGGCATCGATTATCGG	744	52
	OXA-48-F	GAGCACTTCTTTTGTGATGGC		
IS*Aba*1 + bla_{OXA-23}	IS-F	GTGTCATAGTATTCGTCG	875	50
	OXA23-R	ATTTCTGACCGCATTTCCAT		
IS*Aba*1 + bla_{OXA-51}	IS-F	GTGTCATAGTATTCGTCG	359	50
	OXA51-R	CAAGGCCGATCAAAGCATTA		
bla_{IMP}	IMP-F	GGAATAGAGTGGCTTAAYTC	232	50
	IMP-R	TCGGTTTAAYAAAACAACCACC		

*bla*VIM	VIM-F	GATGGTGTTTGGTCGCATA	390	50
	VIM-R	CGAATGCGCAGCACCAG		
*bla*SPM	SPM-F	AAAATCTGGGTACGCAAACG	271	52
	SPM-R	ACATTATCCGCTGGAACAGG		
*bla*GIM	GIM-F	TCGACACACCTTGGTCTGAA	477	50
	GIM-R	AACTTCCAACTTTGCCATGC		
*bla*SIM	SIM-F	TACAAGGGATTCGGCATCG	570	50
	SIM-R	TAATGGCCTGTTCCCATGTG		
*bla*NDM	NDM-F	CACCTCATGTTTGAATTCGCC	984	52
	NDM-R	CTCTGTCACATCGAAATCGC		
*bla*KPC	KPC-F	CGTCTAGTTCTGCTGTCTTG	798	50
	KPC-R	CTTGTCATCCTTGTTAGGCG		
*arm*A	*arm*A-F	ATTCTGCCTATCCTAATTGG	315	56
	*arm*A-R	ACCTATACTTTATCGTCGTC		
*rmt*A	*rmt*A-F	CTAGCGTCCATCCTTTCCTC	635	56
	*rmt*A-R	TTTGCTTCCATGCCCTTGCC		
*rmt*B	*rmt*B-F	ATGAACATCAACGATGCCCT	769	56
	*rmt*B-R	CCTTCTGATTGGCTTATCCA		
*rmt*C	*rmt*C-F	CGAAGAAGTAACAGCCAAAG	711	56
	*rmt*C-F	ATCCCAACATCTCTCCCACT		
*rmt*D	*rmt*D-F	CGGCACGCGATTGGGAAGC	401	51
	*rmt*D-R	CGGAAACGATGCGACGAT		
*rmt*E	*rmt*E-F	ATGAATATTGATGAAATGGTTGC	818	46
	*rmt*E-R	TGATTGATTTCCTCCGTTTTTG		
*rmt*F	*rmt*F-F	GCGATACAGAAAACCGAAGG	589	50
	*rmt*F-R	ACCAGTCGGCATAGTGCTTT		
aacC1	*aacC1-F*	ATGGGCATCATTCGCACATGTAGG	456	60
	aacC1-R	TTAGGTGGCGGTACTTGGGTC		
aadA1	*aadA1-F*	ATGAGGGAAGCGGTGATCG	254	54

	aadA1-R	TTATTTGCCGACTACCTTGGTG		
aadB	*aadB-F*	ATGGACACAACGCAGGTCGC	524	54
	aadB-R	TTAGGCCGCATATCGCGACC		
aphA1	*aphA*1-F	CAACGGGAAACGTCTTGCTC	455	50
	*aphA*1-R	ATTCGTGATTGCGCCTGAG		
aphA6	*aphA6-F*	ATGGAATTGCCCAATATTATTC	797	50
	aphA6-R	TCAATTCAATTCATCAAGTTTTA		
*tet*A	tetA-F	GTGAAACCCAACATACCCC	888	50
	tetA-F	GAAGGCAAGCAGGATGTAG		
*tet*B	tetB-F	CCTTATCATGCCAGTCTTGC	774	50
	tetB-R	ACTGCCGTTTTTTCGCC		
*sul*1	*sul*1-F	CGGCGTGGGCTACCTGAACG	433	58
	*sul*1-R	GCCGATCGCGTGAAGTTCCG		
*sul*2	*sul*2-F	GCGCTCAAGGCAGATGGCATT	293	58
	*sul*2-R	GCGTTTGATACCGGCACCCGT		
adeB	adeB-F	TTAACGATAGCGTTGTAACC	541	50
	adeB-R	TGAGCAGACAATGGAATAGT		
adeG	adeG-F	TTCATCTAGCCAAGCAGAAG	468	50
	adeG-R	GTGTAGTGCCACTGGTTACT		
adeJ	adeJ-F	ATTGCACCACCAACCGTAAC	453	50
	adeJ-R	TAGCTGGATCAAGCCAGATA		
abeD	abeD-F	ATTCAGACCTCTAAGCTCATCCACCAAACT	930	50
	abeD-R	AAAGTTTTCGAAAGTTGAATAATTTTTACT		
adeT1	adeT1-F	TAGAGAATTCGTGTTTGACCCCATTGGTAA	915	55
	adeT1-R	CGGCGGATCCTTATTCATCGTTTAATGCAC		
adeT2	adeT2-F	TAGAGAATTCATGGGTAGTACCTCAGGTAT	717	55
	adeT2-R	CGGCGGATCCTTATCTACTCATTGGACATT		
abeM	abeM-F	AAGTCTTTATTGCCGCACAC	361	50
	abeM-R	ATCGGTGCCTGAGTATCTTG		

amvA	amvA-F	CACTGATTATGGCGGGTTTG	163	52
	amvA-R	CCTAAGACAGCACGAGAAGC		
qacE	qacE-F	CCCGAATTCATGAAAGGCTGGCTT	350	55
	qacE-R	TAAGCTTTCACCATGGCGTCGG		
qacEΔ1	qacEΔ1-F	TAGCGAGGGCTTTACTAAGC	300	52
	qacEΔ1-R	ATTCAGAATGCCGAACACCG		
aceI	aceI-F	GTGTTTATGGCAGTGGTTTCA	148	50
	aceI-R	CAGTCGCAATCAGCAAACCA		
